# Complex *TP53* Alterations with del(17p) and del(13q) in Chronic Lymphocytic Leukemia: Clinical Implications from a Case-Based Review

**DOI:** 10.3390/ijms27135843

**Published:** 2026-06-28

**Authors:** Beata Balla, Andrei Crauciuc, Erzsebet Lazar, Claudia Bănescu

**Affiliations:** 1 Department of Medical Genetics, George Emil Palade University of Medicine, Pharmacy, Science and Technology of Târgu Mureș, 540139 Târgu Mureș, Romania; beakardos@gmail.com (B.B.); claudia.banescu@gmail.com (C.B.); 2 Genetics Laboratory, Center for Advanced Medical and Pharmaceutical Research, George Emil Palade University of Medicine, Pharmacy, Science and Technology of Târgu Mureș, 540139 Târgu Mureș, Romania; 3 Medical Genetics Laboratory, Emergency County Hospital of Târgu Mureș, 540136 Târgu Mureș, Romania; 4 Department of Internal Medicine, George Emil Palade University of Medicine, Pharmacy, Science and Technology of Târgu Mureș, 540139 Târgu Mureș, Romania

**Keywords:** CLL, *TP53*, CNV

## Abstract

Chronic lymphocytic leukemia (CLL) is a genetically heterogeneous disease in which *TP53* alterations represent major adverse prognostic factors; this study aims to describe the clinical implications of complex *TP53* disruption in a rare case context. Molecular and cytogenetic profiling was performed using MLPA for copy number variations and targeted next-generation sequencing for mutation detection, following DNA extraction from peripheral blood and standardized bioinformatic analysis pipelines. The patient exhibited concomitant del(13q14) and del(17p13), alongside two pathogenic *TP53* mutations, indicating functional inactivation of p53 and a high-risk genomic profile; despite this, treatment with a Bruton’s tyrosine kinase inhibitor resulted in significant hematological improvement within six months. These findings highlight that adverse *TP53* alterations override favorable cytogenetic markers and emphasize the necessity of comprehensive genomic testing to guide prognosis and personalized therapy in CLL.

## 1. Literature Review

### 1.1. Chronic Lymphocytic Leukemia

Chronic lymphocytic leukemia (CLL) is probably the most common adult leukemia and is characterized by the continuous accumulation of immunologically incompetent lymphocytes in the blood, lymph nodes, bone marrow, and spleen [1,2]. CLL accounts for approximately 25–30% of all leukemia cases in Western countries [3,4], with more than 100,000 incident cases and over 40,000 deaths reported globally in 2019 [4,5]. Epidemiological data show that CLL incidence increases sharply with advancing age, reaching its highest levels in elderly populations [6]. A pronounced sex disparity is also well documented, with men exhibiting nearly twice the incidence observed in women [2,7]. In addition, significant geographic differences exist: although CLL represents the predominant adult leukemia in Western populations, it remains relatively rare in Asian countries, and its incidence remains low even among Asian individuals who immigrate to Western regions [2,7,8,9,10].

Additional evidence supporting the influence of ethnicity on CLL risk comes from epidemiological studies in the Middle East. Such findings indicate that ethnic background, rather than geographical proximity or shared environmental exposures, may be a more critical determinant of CLL risk [9]. This pattern suggests that inherited rather than environmental factors might play a crucial role in susceptibility to disease [4,10].

CLL is defined by the clonal expansion and accumulation of mature, typically CD5-positive B lymphocytes within the peripheral blood, lymph nodes, bone marrow, and spleen [11,12,13]. Recent findings indicate that the capacity to generate CLL-like clonal B cells may originate as early as the hematopoietic stem cell (HSC) stage, suggesting that the initial leukemogenic event could arise in multipotent, self-renewing HSCs rather than in committed B-cell precursors [14]. Understanding of the leukemogenic process has advanced substantially in recent years. Large cohort studies detailing the genomic landscape of CLL demonstrate that disease initiation frequently involves broad chromosomal aberrations—such as deletion 13q, deletion 11q, or trisomy 12 followed by the acquisition of additional gene mutations that contribute to disease progression and more aggressive clinical behavior [15,16,17].

### 1.2. Cytogenetic Abnormalities in CLL

Cytogenetic abnormalities represent a defining feature of chronic lymphocytic leukemia (CLL) and play a central role in disease pathogenesis, prognosis, and therapeutic decision-making. The most common recurrent chromosomal abnormalities include del(13q), del(11q), trisomy 12, and del(17p), while less frequent lesions comprise gains of 2p and 8q, as well as deletions of 8p and 15q [18,19]. Among these genomic alterations, *TP53* abnormalities, including both del(17p) and *TP53* mutations, carry the greatest prognostic significance and are consistently associated with inferior clinical outcomes, regardless of whether they occur independently or concurrently [18,20].

Deletion of chromosome 13q14 is the most frequent cytogenetic abnormality in CLL, being detected in approximately 55% of patients [21,22]. When present as the sole genetic lesion, del(13q14) is generally associated with a favorable prognosis and a more indolent disease course. The minimally deleted region contains several tumor suppressor elements, including the microRNAs miR-15a and miR-16-1, whose loss contributes to leukemogenesis through dysregulation of apoptosis-related pathways [23,24,25]. Experimental studies have further demonstrated the biological relevance of this region, as mice harboring targeted deletions of the miR-15a/16-1 cluster and the neighboring *DLEU2* locus develop monoclonal B-cell lymphocytosis, CLL-like disease, and lymphoma, supporting their critical role in CLL pathogenesis [7,24,26].

Deletion of chromosome 11q is observed in approximately 10% of patients with early-stage disease and up to 25% of treatment-naive patients with advanced-stage CLL [27]. This abnormality frequently involves the 11q23 region, which contains the *ATM* gene, a key regulator of the DNA damage response pathway. Clinically, del(11q) is associated with extensive lymphadenopathy, more aggressive disease progression, and reduced overall survival [28]. Notably, some of the adverse prognostic impacts traditionally associated with del(11q) appear to be mitigated by modern chemoimmunotherapy and targeted treatment approaches [7].

Trisomy 12 is detected in approximately 10–20% of CLL cases and represents one of the most common chromosomal gains in the disease. Despite its frequency, the biological mechanisms underlying trisomy 12-associated leukemogenesis remain incompletely understood. Similarly, its prognostic significance remains controversial, with studies reporting heterogeneous clinical outcomes among affected patients [29,30].

Deletion of chromosome 17p occurs in approximately 5–8% of newly diagnosed CLL patients and becomes increasingly prevalent in relapsed or refractory disease [28]. This lesion typically encompasses the 17p13 region, which harbors the *TP53* tumor suppressor gene. Loss of *TP53* function profoundly impairs the cellular response to DNA damage, resulting in resistance to conventional chemotherapy and poorer clinical outcomes [30,31,32]. *TP53* mutations are present in approximately 5–15% of newly diagnosed patients, with their frequency increasing substantially during disease progression and after exposure to therapy [33,34]. More than 80% of CLL cases carrying del(17p) also harbor a mutation in the remaining *TP53* allele, leading to biallelic *TP53* disruption [35]. Although less common in the absence of del(17p), *TP53* mutations alone confer a similarly adverse prognostic impact, including reduced response to therapy and shorter overall survival [31,36]. Furthermore, *TP53* abnormalities are strongly associated with increased genomic complexity, supporting the concept that defective DNA damage response mechanisms contribute to genomic instability and clonal evolution in CLL [7].

### 1.3. The TP53 Gene in CLL

**Figure 1:**
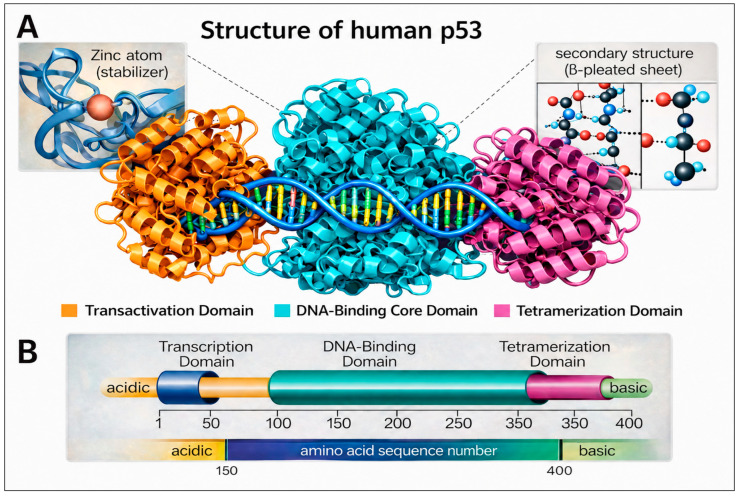
Structure of the human p53 protein. (**A**): Three-dimensional structure of the p53 tetramer bound to DNA. The DNA-binding core domain (cyan) mediates sequence-specific interaction with target genes and contains the majority of pathogenic *TP53* mutations identified in human cancers. The transactivation domain (orange) is responsible for the recruitment of transcriptional machinery and interaction with regulatory proteins. The tetramerization domain (pink) enables the formation of the active p53 tetramer required for efficient DNA binding and transcriptional activity. Zinc ions contribute to stabilization of the DNA-binding domain structure. (**B**): Linear organization of the p53 protein, consisting of an N-terminal transactivation domain (amino acids 1–61), a central DNA-binding domain (amino acids 94–292), a tetramerization domain (amino acids 325–356), and a C-terminal basic regulatory region (amino acids 363–393). Most cancer-associated *TP53* mutations cluster within the DNA-binding domain, resulting in loss of tumor suppressor function and impaired cellular response to DNA damage.

*TP53*, the most frequently mutated gene across human cancers, is a key regulator of genomic stability, and its disruption is strongly linked to unfavorable clinical outcomes. Somatic *TP53* mutations occur in a large proportion of tumors and are typically associated with adverse prognosis due to the loss of p53’s critical tumor-suppressive functions [37,38]. The wild-type p53 protein (Figure 1) serves as a genomic guardian, halting the cell cycle to allow DNA repair or inducing apoptosis when damage is irreparable [38]. Once *TP53* becomes inactivated, these mechanisms fail, leading to genomic instability, accumulation of mutations, and tumorigenesis [39]. Depending on the variant, *TP53* mutations can act through loss-of-function, dominant-negative, or gain-of-function mechanisms. Germline *TP53* mutations are responsible for Li–Fraumeni syndrome (LFS), a hereditary predisposition to multiple early-onset cancers [40,41].

**Figure 2:**
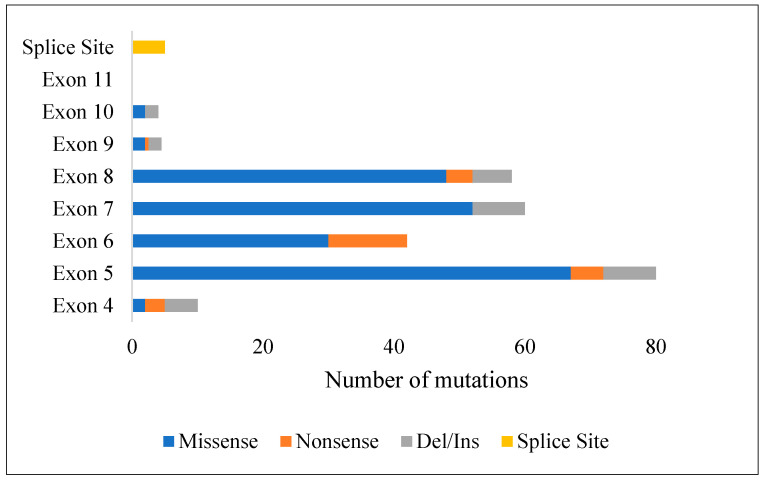
Distribution of *TP53* mutations in patients with CLL (adapted after Thorsten Zenz et al. [45]).

Alterations of *TP53* in chronic lymphocytic leukemia (CLL) were first documented in the early 1990s and have since been recognized as highly prevalent and clinically relevant [42,43]. Most *TP53* defects in CLL occur as deletions of the p arm of chromosome 17 (17p13.1) encompassing the *TP53* locus, frequently accompanied by a missense mutation in the remaining allele, resulting in biallelic inactivation. The distribution of *TP53* mutations in CLL patients is represented in Figure 2 [18,31]. The loss of p53 activity drives leukemic progression by promoting cell survival and resistance to therapy.

Interestingly, deletions involving 17p often extend to neighboring genes such as *POLR2A* and *EIF5A*, further contributing to tumorigenesis and opening potential avenues for synthetic-lethal therapeutic targeting [44].

Thorsten Z et al. reported missense *TP53* variants in about 74% of cases, followed by small deletions/insertions (20%), nonsense (4%) and splice site (2%) mutations, most clustered within the DNA-binding domain (exons 5–8) as seen in Figure 2 [45,46]. Similar distribution of *TP53* mutations has been described by Bertossi et al. in a cohort of over 1300 patients [47]. Among the well-characterized variants, *TP53*:p.Cys238Phe (c.713G>T)—within the zinc-binding region—causes structural destabilization and exhibits a dominant-negative effect. This rare variant is absent from population databases and classified as pathogenic/likely pathogenic in multiple clinical repositories [48]. In contrast, *TP53*:c.574C>T (p.Gln192Ter) introduces a premature stop codon leading to truncation and loss of function through nonsense-mediated decay. While pathogenic in germline Li–Fraumeni syndrome, its role in somatic hematologic malignancies like CLL remains uncertain [45,49,50].

Missense mutations in *TP53* represent the most frequent genetic alteration across human cancers. Such mutations frequently disrupt the DNA-binding domain of *TP53*, leading to defective transcriptional activation of genes regulating DNA repair, apoptosis, and cell cycle arrest [51,52]. Despite extensive research, the exact structural and functional consequences of many *TP53* variants remain incompletely understood, leaving gaps in our knowledge of their clinical significance. The prevalence of *TP53* aberrations varies with disease stage and *IGHV* mutational status. They occur in 5–10% of newly diagnosed patients, 10–20% in those starting first-line therapy, and rise to 50% in relapsed or Richter transformation cases [53]. In CLL, del(17p) is frequently accompanied by *TP53* mutations, while isolated deletions are rare and isolated mutations are more common [54,55,56,57]. The combination of deletion and mutation typically predicts the poorest survival, even in the era of targeted agents such as BTK inhibitors (e.g., ibrutinib) [55].

### 1.4. Detection of TP53 Mutations

Detection of *TP53* mutations may be performed using Sanger, Nanopore or next-generation sequencing (NGS). At a minimum, the analysis should include exons 4 through 9, including the corresponding splice junctions, although the optimal coverage extends from exons 2 to 11 [53,58,59]. In accordance with ERIC (European Research Initiative on CLL) recommendations regarding sensitivity thresholds, only mutations identifiable by Sanger sequencing or, when using NGS, those with a variant allele frequency exceeding 10% should be reported [59,60]. The introduction of NGS into routine diagnostics has enabled the identification of *TP53* variants present at levels below the detection threshold of Sanger sequencing, traditionally set at approximately 10% variant allele frequency (VAF). For variants detected at <10% VAF, the recommended terminology includes “low-burden,” “minor-clone,” “low-VAF,” or “low-level” mutations. The term “subclonal” should be avoided in this context, as it conventionally refers to mutations present in only a subset of the tumor population, in contrast to “clonal” variants. Importantly, determining whether a *TP53* mutation is clonal or subclonal requires knowledge of the tumor cell fraction in the analyzed sample and the copy number status of the *TP53* locus, parameters that are typically not available in standard molecular diagnostic workflows [56,61,62].

### 1.5. Cooccurrence of del17p and TP53 Mutations

Using ultra-deep next-generation sequencing (NGS), Bomben et al. (2023) [55] further investigated the prognostic interaction between *TP53* mutations and deletions. Their study categorized patients as: wild-type (non-del17p/non-*TP53* mutated), del(17p) only, *TP53*-mutated only, or both del(17p) and *TP53* mutated. Only the group with combined aberrations exhibited significantly shorter overall and progression-free survival, underscoring the additive prognostic impact of complex *TP53* inactivation [55].

In a large study by László et al. (2024) [63], where del(17p) status was assessed using FISH in 795 patients, this abnormality was detected in 9.2% (73/795) of cases. Among individuals with del(17p), 75.3% (55/73) also harbored a *TP53* mutation, 12 classified as low-burden and 43 as high-burden, indicating frequent biallelic *TP53* inactivation. Conversely, 39.9% (55/138) of all *TP53*-mutated cases demonstrated a concomitant del(17p). Sixteen patients carried multiple *TP53* mutations (2–5 per case) alongside del(17p), consistent with a genetically heterogeneous subclonal architecture. Notably, del(17p) occurred more often in patients with high-burden *TP53* mutations than in those with low-burden variants (52% vs. 22%, *p* = 0.0007). In contrast, most patients with isolated low-burden *TP53* mutations (78.2%, 43/55) lacked del(17p) [30,61].

Zenz et al. (2008) [33] analyzed 126 well-characterized CLL patients using direct sequencing and DHPLC (Denaturing High-Performance Liquid Chromatography) to detect *TP53* mutations across exons 2–11. Consistent with prior observations, *TP53* mutations were found in the majority of cases with del(17p) (81%), whereas only 4.5% of patients without del(17p) carried a *TP53* mutation [33]. Patients harboring *TP53* mutations (*n* = 18) demonstrated significantly inferior survival (*p* = 0.002), a difference that became even more pronounced when survival was calculated from the time of mutation detection (6.8 vs. 69 months, *p* < 0.001). Outcomes were similarly poor across subgroups: with both del(17p) and *TP53* mutation (7.6 months, *n* = 13), *TP53* mutation alone (5.5 months, *n* = 5), and del(17p) alone (5.4 months, *n* = 3) [33]. The coexistence of both defects was strongly correlated with inferior prognosis, establishing *TP53* status as a fundamental prognostic biomarker in CLL. Multivariate analysis confirmed that *TP53* mutation status (HR 3.71) remained an independent predictor of adverse prognosis, irrespective of Rai stage, *IGHV* status, or del(11q)/del(17p). Serial sampling indicated progressive clonal expansion during chemotherapy, suggesting potential treatment-driven selection. Together, these findings reinforce that once acquired, *TP53* mutations confer markedly poor survival in CLL [33,36,64]. Consequently, assessment of *TP53* deletions and mutations has become routine in clinical diagnostics, while *TP53* mutation databases such as ClinVar, IARC, and OncoKB are essential for validating and interpreting tumor variants. CLL displays considerable subclonal heterogeneity, often with multiple *TP53* mutations at low variant allele frequencies (VAFs), reflecting complex clonal architecture [33,53,65].

The reliability of NGS for detecting low-VAF (<10%) *TP53* variants has been debated. A multicenter evaluation of 41 laboratories demonstrated sensitivities of 85.6%, 94.5%, and 94.8% at VAF cutoffs of 1%, 2%, and 3%, respectively. Although false positives were rare above 2% VAF, distinguishing true mutations from background noise below this threshold proved challenging. Clinically, in a cohort of 1092 CLL cases not treated with targeted agents, low-VAF *TP53* mutations were associated with shorter time-to-second-treatment (TTST) and overall survival (OS) compared with *TP53*–wild-type patients, with risk increasing proportionally with VAF. The adverse effect was attenuated in patients receiving targeted therapy in ≥2 lines, whereas high-VAF *TP53* mutations continued to predict inferior survival [66,67].

Allelic burden analysis in a study by Lazarian et al. (2019) [68] further revealed that 264 of 573 (46%) detected *TP53* mutations had a VAF < 10%. Although most occurred in polymutated cases, 21% of patients carried exclusively low-VAF mutations—findings that would have been completely missed by conventional sequencing. Multiclonality was common: among 343 patients, 113 (33%) harbored multiple pathogenic *TP53* variants (2–11 per patient). Long-range sequencing and computational analysis demonstrated that these mutations mapped to different alleles, indicating substantial intratumoral heterogeneity and strong selective pressure for *TP53* loss of function. Null mutations were rarely isolated events; 87% of null variants occurred in highly poly-mutated cases (four or more mutations). Variant size decreased as the number of mutations increased, and low-VAF variants predominated in multiclonal contexts. Multiclonal *TP53* mutations were more frequent in previously treated patients (41% vs. 10%), suggesting therapy-driven clonal selection, though their pre-treatment existence remains unresolved. Notably, the presence or absence of del(17p) did not correlate with the number of *TP53* mutations [68].

Limited large-scale data exist on the correlation between NGS-detected *TP53* mutations and del(17p) by FISH. The largest cohort to date (*n* = 2332) found that *TP53* aberrations (mutation and/or del(17p)) were present in 13.7% of cases. NGS identified 429 mutations in 303 patients (13%), of which 79% were high-burden (≥10% VAF) and 21% were low-burden. *TP53* mutations without del(17p) were detected in 159 cases (49 low-burden), while 144 patients carried both aberrations (16 low-burden). Only 17 patients (0.7%) exhibited del(17p) in the absence of a detectable *TP53* mutation. These findings confirm that validated NGS workflows can detect the vast majority of *TP53* abnormalities and are essential for avoiding missed low-burden variants. Overall, del(17p) defines a distinct genomic subgroup, and clonal *TP53* mutations, deletions involving 3p, 4p, or 9p, as well as genomic complexity, are strongly associated with inferior survival outcomes [69].

To date, no documented CLL cases have described the concomitant presence of two distinct *TP53* mutations alongside del(17p) and del(13q). Such a combination likely represents a complex genomic landscape of extreme clonal heterogeneity, contributing to aggressive disease behavior, resistance to therapy, and poorer clinical outcomes.

## 2. Materials and Methods

The present study has been approved by the Ethics Committee of the Emergency County Hospital of Târgu Mureș, by decision no. 8922/18.04.2024. After receiving informed consent from the patient, 2 mL of whole blood sample was collected on EDTA, and DNA was extracted using the NucleoSpin Blood Mini kit (Macherey-Nagel, Düren, Germany). DNA concentration was measured using Qubit™ Flex Fluorometers (Invitrogen™, Carlsbad, CA, USA), and subsequent dilutions were performed according to the manufacturer’s recommendation. FISH analysis was not available for this patient; therefore, copy number variation testing has been performed by MLPA due to its cost-effective method performed in our lab, and to the capacity for detecting multiple deletions and duplications of the chromosomal regions recurrently imbalanced in CLL. SALSA^®^ MLPA^®^ Probemix P038 CLL-2 (MRC Holland, Amsterdam, the Netherlands) has been used, available from MRC Holland, and Coffalyser.Net v.250317.1029 data analysis software. The SALSA MLPA Probemix P038 CLL-2 is designed to detect deletions and duplications in genomic regions frequently altered in chronic lymphocytic leukemia, including 10q23 (*PTEN*), 11q (*ATM*), chromosome 12, 13q14 (*RB1*, *DLEU1/2*), 14q, 17p13 (*TP53*), and chromosome 19. In addition, this probemix enables the detection of specific point mutations, namely *NOTCH1* c.7541-7542delCT (p.P2514Rfs*4), *SF3B1* c.2098A>G (p.K700E), and *MYD88* c.794T>C (p.L265P, also referred to as p.L252P). Next-generation sequencing was performed using the ONCOMINE myeloid panel.

The DNA library was prepared using an automated workflow on the Ion Chef System (Thermo Fisher Scientific) and Oncomine Myeloid Chef DNA (Ion Torrent, Thermo Fisher Scientific, Waltham, MA, USA), which covers 23 hotspot genes and 17 full genes. The library was loaded on Ion 530 Chip Kit (Thermo Fisher Scientific, Waltham, MA, USA) and sequenced on the Ion S5. The data were analyzed using the Ion Reporter 5.20 platform and Oncomine Variants 5.20. Sequencing reads were aligned to the hg19 human reference genome (GRCh37), and the variants were reported according to hg19 coordinates.

## 3. Case Presentation

We report the case of a 79-year-old male patient referred to the Department of Medical Genetics for comprehensive molecular and cytogenetic evaluation following a confirmed clinical and hematological diagnosis of chronic lymphocytic leukemia (CLL). The referral aimed to define the patient’s molecular risk profile, refine prognostic stratification, and inform personalized therapeutic management, in accordance with current international guidelines.

Peripheral blood analysis demonstrated a white blood cell (WBC) count of 60.36 × 10^9^/L (reference range: 4–10 × 10^9^/L) with lymphocytes accounting for 87.6% of total leukocytes (reference range: 20–45%). Peripheral blood immunophenotyping revealed 73% lymphoid elements with the following antigenic profile: CD19+, CD20+, CD5+ (32%), CD22+, CD10-, CD200+, CD79b+, CD27+ (12.5%), LAIR1+, CD81+, CD43+ (6%), CD23+ (47%), FMC-, kappa+. The patient’s hemoglobin and LDH level, as well as platelet count, were within normal limits, and there was no biochemical or clinical evidence of autoimmune cytopenia, hepatosplenomegaly, or lymphadenopathy.

To characterize the disease’s genetic landscape, the patient underwent a comprehensive molecular workup, including copy number variation (CNV) and sequence variant analysis.

1. Copy Number Variation (CNV) Testing: CNV assessment was performed using Multiplex Ligation-dependent Probe Amplification (MLPA) with the MRC Holland P038 kit, designed to detect recurrent CLL-associated cytogenetic abnormalities. This assay screens for common chromosomal imbalances involving 13q14, 11q22.3, 12q15, and 17p13.1.

The MLPA analysis identified two significant alterations:

- A heterozygous deletion in 13q14 affecting all markers included in the kit for this region (*RB1*, *KCNRG*, *MIR15A*, *DLEU1*, and *ATP7B* genes)—Final Ratio: 0.47–0.61. Del13q14 is the most frequent aberration in CLL, typically associated with a favorable prognosis when present as an isolated event.
- A heterozygous deletion in 17p13, affecting the *TP53* locus (all markers included in the kit—Final Ratio: 0.35–0.67). This finding is recognized as one of the strongest independent adverse prognostic markers, conferring chemoresistance, shorter time to progression, and reduced overall survival.
- *NOTCH1* c.7541-7542delCT (p.P2514Rfs*4), *SF3B1* c.2098A>G (p.K700E), and *MYD88* c.794T>C (p.L265P, also referred to as p.L252P) mutations included in the kit, were negative.

2. Next-Generation Sequencing (NGS) Analysis: To further characterize potential molecular lesions, targeted NGS was performed using the ONCOMINE Myeloid panel, which screens for mutations in key genes involved in myeloid and lymphoid malignancies. Sequencing metrics demonstrated robust technical performance, with 1,906,085 mapped reads, 94.48% on-target reads, a mean depth of 3434, and base coverage uniformity of 97.72%. Importantly, 99.46% of target bases achieved ≥100× coverage, and 98.70% exceeded 500× coverage, confirming high-confidence detection of subclonal variants. Amplicon performance amplification was homogeneous across all 526 targets, with 99.43% of amplicons exceeding 100 reads and 96.77% showing no strand bias. The Ti/Tv ratio (2.474) and dbSNP concordance (0.986) were consistent with a low background error rate and high variant calling fidelity. Given the semiconductor-based detection chemistry of Ion Torrent technology, particular attention was given to homopolymer-associated insertion/deletion artifacts. The high uniformity of coverage, absence of amplicon dropout, balanced strand representation, and high AQ20 base proportion collectively reduce the likelihood of systematic sequencing artifacts. Variant calls were further filtered using coverage depth, strand bias metrics, and quality score thresholds within the somatic workflow.

Sequencing results revealed two distinct pathogenic variants in *TP53*:

- *TP53*:c.713G>T (p.Cys238Phe) VAF 12.45%, a somatic, missense, heterozygous mutation within the DNA-binding domain, substituting cysteine with phenylalanine. This alteration disrupts the zinc-binding site, leading to conformational instability of the p53 protein and resulting in loss of transcriptional activity.
- *TP53*:c.574C>T (p.Gln192Ter) VAF 14.8%, a somatic, nonsense heterozygous mutation introducing a premature stop codon at amino acid position 192, leading to protein truncation, effectively resulting in loss of function.

With a mean coverage depth exceeding 3400× and >98% of bases covered at ≥500×, the theoretical analytical sensitivity supports reliable detection of somatic variants at variant allele frequencies (VAF) of approximately 3–5%, assuming locus-specific depth ≥500× and balanced strand representation. At coverage levels >1000×, stochastic sampling error is markedly reduced, and binomial confidence intervals narrow substantially, allowing discrimination between true low-frequency variants and background noise. Considering the observed sequencing quality parameters and uniformity, the effective limit of detection in this sample is estimated to be below 5% VAF. The present testing approach cannot establish the allelic phase of the two variants; therefore, it remains unclear whether they occur in cis (on the same allele) or in trans (on different alleles).

The coexistence of del(17p13) and *TP53* disruption through two distinct sequence variants indicates a complex genotype and is considered a high-risk molecular signature, associated with rapid disease progression and poor response to conventional chemoimmunotherapy regimens such as fludarabine, cyclophosphamide, and rituximab (FCR). Considering the presence of *TP53* disruption (del(17p13) and *TP53* mutations), the patient was started on Ibrutinib, a Bruton’s tyrosine kinase (BTK) inhibitor, as first-line targeted therapy, given its established efficacy in *TP53*-aberrant CLL. At 3-month follow-up, the patient presented a WBC count of 20.19 × 10^9^/L (reference range: 4–10 × 10^9^/L) with lymphocytes accounting for 51% of total leukocytes (reference range: 20–45%), and an elevated LDH of 262 U/L (reference range: 135–225 U/L). At 6-month follow-up, WBC count decreased to 8.94 × 10^9^/L with slightly elevated lymphocytes, accounting for 47.3% of total leukocytes, while LDH level was not determined. The patient demonstrated a favorable clinical and hematological response, with normalization of WBC count and lymphocyte percentage within a 6-month period of treatment, consistent with the expected therapeutic profile of Ibrutinib in this high-risk molecular subgroup.

## 4. Discussion

The prognostic stratification of chronic lymphocytic leukemia (CLL) has evolved substantially over the past two decades, largely owing to the integration of cytogenetic and molecular findings into clinical practice. The hierarchical model proposed by Döhner et al. (2000) [19] remains a cornerstone for genetic risk assessment in CLL, relying on fluorescence in situ hybridization (FISH) for detection of the most recurrent chromosomal abnormalities: 13q14 deletion [del(13q)], 17p13 deletion [del(17p)], 11q22 deletion [del(11q)], and trisomy 12 (+12) (27). In this framework, isolated del(13q) confers the most favorable prognosis, often associated with indolent disease, whereas del(17p) is strongly correlated with shorter survival and poor response to chemotherapy [19,60].

Over the past decade, studies have demonstrated that the cytogenetic complexity itself—beyond individual aberrations— carries important prognostic implications. In particular, Nguyen-Khac et al. (2023) emphasized the negative impact of a complex karyotype (CK), defined as the presence of at least three chromosomal abnormalities, on disease progression and therapeutic outcomes [70]. Although our patient’s cytogenetic profile does not strictly meet the CK definition, the simultaneous presence of multiple high-risk lesions (dual *TP53* mutations plus del(17p)) reflects an underlying genomic instability consistent with this observation [70,71,72].

From a molecular perspective, *TP53* aberrations are among the most deleterious genomic events in CLL. These alterations can occur through point mutations, small insertions/deletions, or large-scale deletions encompassing the *TP53* locus. They may arise independently or in combination, resulting in either monoallelic or biallelic inactivation of the gene. The latter scenario, observed in our patient, is particularly detrimental and is associated with aggressive clinical behavior and resistance to standard therapy [18,53,73,74].

Multiple reports have confirmed that *TP53* mutations and deletions are the strongest independent prognostic factors in CLL, predicting resistance to chemoimmunotherapy (CIT) and shorter progression-free and overall survival [53]. Consequently, the detection of *TP53* aberrations, even in the absence of del(17p), mandates a therapeutic shift toward targeted therapies such as Bruton’s tyrosine kinase (BTK) inhibitors (e.g., ibrutinib, acalabrutinib) or BCL2 inhibitors (e.g., venetoclax), rather than traditional CIT regimens. In our case, the presence of combined *TP53* lesions directly influenced the therapeutic decision, leading to the initiation of ibrutinib as first-line treatment, in accordance with international recommendations [75,76].

Notably, Zenz et al. (2010) demonstrated that *TP53* mutations alone—without concomitant del(17p)—still predict poor survival and chemoimmunotherapy resistance, underscoring the independent pathogenic impact of these alterations [45]. Similarly, Campo et al. (2018) reported that patients harboring both del(17p) and *TP53* mutations had the worst clinical outcomes, whereas *TP53*-mutated-only or del(17p)-only cases had intermediate risk [18]. The dual disruption observed in our patient exemplifies this high-risk genomic scenario and supports the growing consensus that *TP53* inactivation—regardless of the underlying mechanism—constitutes a distinct biological entity within CLL characterized by rapid disease evolution and inferior prognosis.

In a broader hematologic context, the adverse prognostic value of *TP53* mutations extends beyond CLL. In a comparative study by Stengel et al. (2017) [77], *TP53* mutations alone significantly impacted survival in acute myeloid leukemia (AML; 36 vs. 9 months, *p* < 0.001) and myelodysplastic syndromes (MDS; 65 vs. 19 months, *p* < 0.001), whereas *TP53* deletions had a measurable negative effect in CLL (*p* = 0.008) and MDS (*p* = 0.011) (95). These data underscore the cross-disease consistency of *TP53* loss as a marker of genomic instability and therapy resistance [77].

The co-occurrence of del(17p) and *TP53* mutation generally signifies a combined inactivation, leading to complete functional loss of the p53 protein. Such cases, as described by Campo et al. (2018) [18], exhibit a particularly aggressive clinical phenotype characterized by rapid disease progression, chemo-resistance, and shortened overall survival. In contrast, monoallelic inactivation (via either deletion or mutation alone) is associated with a somewhat milder course, although still worse than *TP53* wild-type CLL. The dual mutation pattern identified in our patient (*TP53*:c.713G>T and *TP53*:c.574C>T), together with del(17p), likely reflects clonal evolution and intra-tumoral heterogeneity, as supported by recent genomic profiling studies demonstrating that multiple *TP53* subclones can coexist within a single patient [65,73].

In our case, *TP53* variants detected at VAF levels of approximately 12–15% fall well above the conservative limit of detection, as supported by sequencing depth and quality metrics. Given the high coverage, minimal strand bias, appropriate Ti/Tv ratio, and absence of significant amplicon imbalance, these variants are unlikely to represent technical artifacts and are consistent with true subclonal somatic events. The combination of high sequencing depth, homogeneous amplicon performance, and stringent bioinformatic filtering provides strong technical support for the biological validity of the identified *TP53* mutations.

Interestingly, our patient also carried a concomitant del(13q14), classically linked to indolent CLL. This combination—high-risk *TP53* defects with low-risk del(13q)—illustrates the complexity of genotype–phenotype correlations in modern CLL genetics [21,78]. While del(13q) alone would suggest a favorable course, the simultaneous presence of *TP53* inactivation effectively negates any protective prognostic effect [21]. This interaction aligns with the hierarchical framework proposed by Döhner et al. (2000), where adverse lesions dominate prognostic interpretation, regardless of coexisting low-risk markers [19].

The patient’s positive response to ibrutinib, manifested by normalization of white blood cell and lymphocyte counts within six months, mirrors findings from recent clinical trials demonstrating that BTK inhibitors can partially overcome *TP53*-associated resistance. However, emerging evidence suggests that even these targeted agents may not fully eliminate high-risk clones, and disease relapse driven by secondary resistance mutations (e.g., BTK or PLCG2) can occur after prolonged therapy [54,79,80].

Mutations in the *SF3B1* gene, which are found in 10% on CLL cases and are associated with worse prognosis, were not observed in our patient.

Limitations of the study: Despite the broader context provided by the literature review, several limitations related to the presented case should be acknowledged. First, the study is based on a single case presentation, limiting the generalizability of the observations to the broader CLL population. Although such reports are valuable for highlighting rare genomic constellations, they cannot establish broader prognostic significance or clinical prevalence. Second, the follow-up period remains relatively short, limiting the ability to assess long-term outcomes, durability of response to targeted therapy, or the eventual emergence of relapse, which is particularly relevant in *TP53*-disrupted disease. Furthermore, the genomic investigation was limited to copy-number variation analysis by MLPA and targeted next-generation sequencing using a predefined panel, which may have underestimated the full mutational and cytogenetic complexity of the leukemia. Additional genomic lesions with prognostic or therapeutic relevance may therefore have remained undetected. *IGHV* mutational status analysis was not available for the presented patient. Finally, serial molecular monitoring was not performed during follow-up, preventing assessment of clonal evolution, persistence of *TP53*-mutated subclones, or acquisition of secondary resistance mechanisms under ibrutinib therapy. Collectively, these limitations highlight the need for extended longitudinal studies and broader genomic characterization to better define the clinical implications of complex *TP53* disruption patterns in CLL.

Table 1 summarizes the results and evolution of several clinical studies investigating *TP53* mutations and CNVs in CLL patients.

Our findings are consistent with previous studies demonstrating the major prognostic impact of *TP53* aberrations in CLL. By integrating mutational and cytogenetic data from a large cohort of 1274 CLL patients, Rossi et al. identified four hierarchical genetic risk groups, with patients harboring *TP53* and/or *BIRC3* abnormalities exhibiting the poorest outcomes, achieving a 10-year overall survival of only 29%. In contrast, patients carrying isolated del(13q14) represented the most favorable prognostic subgroup, with survival rates approaching those of the general population [89]. The adverse prognostic significance of *TP53* disruption has also been confirmed in the era of targeted therapies. Long-term analyses of first-line ibrutinib-based treatment have shown encouraging outcomes in patients with *TP53* aberrations, including del(17p) and *TP53* mutations, with four-year progression-free and overall survival rates of 79% and 88%, respectively. Nevertheless, despite these improvements, *TP53*-defective CLL continues to be associated with an increased risk of disease progression and treatment failure compared with lower-risk genetic subgroups [90].

Importantly, the introduction of Bruton tyrosine kinase (BTK) inhibitors has modified the prognostic impact of several traditional risk factors. Abnormalities such as del(11q) and unmutated *IGHV* status, previously associated with poor outcomes in the chemoimmunotherapy era, appear to have a reduced prognostic impact in patients treated with BTK inhibitors [76]. In contrast, del(17p) and *TP53* mutations remain among the most powerful adverse prognostic markers, even in the context of targeted therapies. For example, in a study by Sivina et al., patients with relapsed or refractory CLL carrying del(17p) and/or *TP53* mutations achieved a median progression-free survival of only 26 months with ibrutinib therapy, compared with 52 months in the overall study population [91].

These observations highlight the critical importance of comprehensive *TP53* assessment, including both *TP53* mutation analysis and evaluation of 17p deletion, before each line of therapy. Accurate identification of *TP53* abnormalities is essential for optimal risk stratification and treatment selection. Furthermore, standardization of sequencing methodologies and variant interpretation remains necessary, particularly for low-frequency subclonal *TP53* mutations whose clinical significance continues to be investigated. Although novel targeted agents such as ibrutinib, idelalisib, and venetoclax have substantially improved outcomes for high-risk patients, *TP53* disruption continues to define a biologically aggressive subgroup requiring careful monitoring and individualized therapeutic strategies [18].

## 5. Conclusions

This case illustrates the prognostic and therapeutic importance of comprehensive genetic profiling in CLL. While del(13q14) in isolation could suggest a favorable outcome, the co-occurrence of del(17p13) and *TP53* mutations reclassifies the patient into a very high-risk group, mandating targeted therapy.

The favorable response to Ibrutinib underscores the value of precision medicine in the management of genetically defined CLL subtypes. Continued follow-up with serial molecular monitoring will be essential to detect potential clonal evolution or emergent resistance mechanisms during ongoing ibrutinib therapy.

## Figures and Tables

**Table 1:** Overview of clinical studies investigating *TP53* mutational status and genomic copy number alterations in CLL.

*TP53* Mutations		del17p	del13q	Other CNVs	Evolution	Patients	Reference *
Single	Multiple						
X		X	X	X		132	[81]
	X	X	X	X	Unfavourable	149	[82]
	X	X	X	X	Unfavourable	489	[16]
		X	X	X	Unfavourable	325	[19]
		X	X	X	Unfavourable	126	[33]
	X	X	X	X	Unfavourable		[21]
		X	X	X		100	[22]
			X	X		50	[83]
			X	X		1	[84]
			X	X		263	[23]
			X	X		60	[24]
	X	X	X	X	Unfavourable		[25]
		X	X	X	Unfavourable		[85]
X		X	X	X		30	[32]
X		X	X	X		44	[35]
	X	X	X	X	Unfavourable		[36]
	X	X	X	X	Unfavourable	683	[43]
X		X	X	X	Unfavourable	328	[45]
		X	X	X	Unfavourable		[46]
	X	X	X	X	Unfavourable	499	[54]
	X	X	X	X	Unfavourable	229	[55]
	X	X	X	X	Unfavourable		[56]
	X	X	X	X	Unfavourable	309	[57]
X		X	X	X		12	[59]
	X	X	X	X	Unfavourable	538	[61]
	X	X	X	X	Unfavourable		[62]
X	X	X	X	X		325	[86]
X	X	X	X	X		290	[87]
X	X	X	X	X	Unfavourable	901	[63]
		X	X	X	Unfavourable	308	[64]
	X	X	X	X	Unfavourable	1092	[67]
	X	X	X	X	Unfavourable	1220	[66]
	X	X	X	X	Unfavourable	683	[68]
	X	X	X	X	Unfavourable	2332	[69]
X		X	X	X	Unfavourable		[72]
X		X	X	X			[74]
		X	X	X	Unfavourable	1148	[77]
X	X	X	X	X	Unfavourable	400	[88]
	X	X	X	X	Unfavourable	84	[79]
	X	X	X	X	Unfavourable	308	[80]

X marks the presence of the genetic anomaly; * the references correspond to the original studies from which the cases were extracted.

## Data Availability

The raw data supporting the conclusions of this article will be made available by the authors on request.

## Abbreviations

The following abbreviations are used in this manuscript:

ATM	Ataxia-telangiectasia mutated
CIT	Chemoimmunotherapy
CLL	Chronic lymphocytic leukemia
CNV	Copy number variation
DDR	DNA damage response
DHPLC	Denaturing high-performance liquid chromatography
HSC	Hematopoietic stem cell
LFS	Li–Fraumeni syndrome
MLPA	Multiplex ligation-dependent probe amplification
NGS	Next-generation sequencing
OS	Overall survival
VAF	Variant allele frequency
